# Equanimity of Mind

**Published:** 1882-07

**Authors:** 


### EQUANIMITY OF MIND,

How health-saving, how dignified, how philosophical! per contra, the *Fiery Folk*, the lapdog sort, how terribly fierce they are, in a moment blazing hot—about nothing! when you come to examine into the merits of the case. Spasmodic people, who take the world by fits and starts; in the skies to-day; to-morrow—in the cellar! every thing, any thing, nothing: doubtless they have their uses, like the insect of an hour, or an atom in the air, but if ever any single individual of this, not innumerous tribe, came to anything, we have yet to arrive at the knowledge of the fact; their more obvious uses are, by misapprehensions, to set people by the ears, to excite family quarrels, and originate ill will among neighbors. Whipper-snappers are they, busy-bodies, never happier than when up to their eyes in other people's business, at the expense of their own; marvellously benevolent towards every body else but their own wives and children. The end of such is to die early, and to die poor; or if they live long, their lives are more a burden than a blessing to the community among whom it was their lot to fall.

Widely different is the aim and end of the man who takes the world calmly, who takes time to make himself *master of the whole fact* before he moves a step; such a man seldom acts wrong, is seldom found in a false position, and is consequently under no necessity of resorting to humiliating quibbles, or even questionable expedients, to extricate him from a predicament; the necessary result being, in the course of years, an abiding impression on his own mind that he has acted right in all things, and being conscious of no wrong, of no quibble, feels always safe, is subject to no unpleasant surprises. Such a man, having no ground for apprehensions, and yet being open to all the enjoyments which other men are, has that stereotyped *equanimity of mind*, which, while it closes the gate against the host of ills which wrong-doing entails on the wicked, opens a door to pleasures innumerable, boundless! These are the men who uniformly "succeed" in life, in any country, and in every clime:—succeed, not merely in the accumulation of money, but in that which is of greater worth, in securing a position in society, and a name, which is the synonyme for all that is solid, manly and good—not only so, the pulses of life beating regularly, they necessarily beat long, for the system is subject to no shocks, is racked by no explosions:—so that they have not only made a fortune and a name, but secured long life to enjoy them, that very enjoyment consolidating both.

This is not a beautiful theory spun out at our own office fire of a bright frosty morning in December, nor yet under the unreliable exhilaration of a cup of tea or a glass of wine, but it is the result of convictions founded on the observation of men and things, confirmed by a *living truth*, in the persons of a whole community of people, whose increasing fewness in the old world, as well as in the new, we on some accounts do sincerely deplore, we mean the "Society of Friends," commonly called *Quakers*. Recent published statistics in England show, that while the average duration of human life is estimated at thirty-three years, that average among these people is fifty-one years, exhibiting thus, this broad practical fact, *that a course of life which promotes an habitual* EQUANIMITY OF MIND, the feature which stands out above all others in the every-day character of Friends in this country as well as in England, *is highly conducive to bodily health and length of days.*

---

### A SICK MIND.

NOT a diseased mind; that might imply something akin to insanity; such is not our meaning. The body may be diseased functionally; the whole machinery is perfect as to parts, no portion is broken or destroyed, but it does not work well,—so we mean as to the mind, when we term it "sick;" it is perfect, it is sound, no decay, but it does not work smoothly, effectively.

